# Supplementary material for: Interacting evolutionary pressures drive mutation dynamics and health outcomes in aging blood
Source: Nat Commun. 2021 Aug 13;12:4921. doi: 10.1038/s41467-021-25172-8 (PMC8363714; doi:10.1038/s41467-021-25172-8)
Supplement: Supplementary file 3 — Reporting Summary [file 41467_2021_25172_MOESM3_ESM.pdf]

nature portfolio

Corresponding author(s): Morris and Awadalla

Last updated by author(s): 7/8/2021

Reporting Summary

Nature Portfolio wishes to improve the reproducibility of the work that we publish. This form provides structure for consistency and transparency in reporting. For further information on Nature Portfolio policies, see our Editorial Policies and the Editorial Policy Checklist.

Statistics

For all statistical analyses, confirm that the following items are present in the figure legend, table legend, main text, or Methods section.

n/a	Confirmed	
☐	☒	The exact sample size (*n*) for each experimental group/condition, given as a discrete number and unit of measurement
☐	☒	A statement on whether measurements were taken from distinct samples or whether the same sample was measured repeatedly
☐	☒	The statistical test(s) used AND whether they are one- or two-sided<br>*Only common tests should be described solely by name; describe more complex techniques in the Methods section.*
☐	☒	A description of all covariates tested
☐	☒	A description of any assumptions or corrections, such as tests of normality and adjustment for multiple comparisons
☐	☒	A full description of the statistical parameters including central tendency (e.g. means) or other basic estimates (e.g. regression coefficient) AND variation (e.g. standard deviation) or associated estimates of uncertainty (e.g. confidence intervals)
☐	☒	For null hypothesis testing, the test statistic (e.g. *F*, *t*, *r*) with confidence intervals, effect sizes, degrees of freedom and *P* value noted<br>*Give P values as exact values whenever suitable.*
☒	☐	For Bayesian analysis, information on the choice of priors and Markov chain Monte Carlo settings
☒	☐	For hierarchical and complex designs, identification of the appropriate level for tests and full reporting of outcomes
☐	☒	Estimates of effect sizes (e.g. Cohen's *d*, Pearson's *r*), indicating how they were calculated

Our web collection on statistics for biologists contains articles on many of the points above.

Software and code

Policy information about availability of computer code

Data collection	All simulations were performed using the SFScode listed here http://sfscode.sourceforge.net/SFS_CODE/index/index.html using the parameters described in the paper.
Data analysis	Following the simulation of data using SFSCode, all training and testing of deep neural networks, analyses and figures were performed in the R statistical programming environment (v.4.0.4). Libraries that are required include plyr v.1.8.6 (32), dplyr v.1.0.5 (33), UpSetR v.1.4.0 (34), Rtsne v.0.15 (35), lsmeans v.2.3.0-0(36), forestmodel v.0.6.2 (37), survival v. 3.2-10 (38), survminer v.0.4.9 (39) and keras v.2.4.0 (40). Figures were generated using ggplot2 v.3.3.3 (41), ggpubr v.040 (42), ggplotify v.0.0.7 (43) and patchwork v.1.1.1 (44). The reproducible code is available at https://github.com/ kimskead/popgenArch.

For manuscripts utilizing custom algorithms or software that are central to the research but not yet described in published literature, software must be made available to editors and reviewers. We strongly encourage code deposition in a community repository (e.g. GitHub). See the Nature Portfolio guidelines for submitting code & software for further information.

Data

Policy information about availability of data

All manuscripts must include a data availability statement. This statement should provide the following information, where applicable:

- Accession codes, unique identifiers, or web links for publicly available datasets
- A description of any restrictions on data availability
- For clinical datasets or third party data, please ensure that the statement adheres to our policy

All data included here was collected and published in a previous Nature Publications (Abelson et al. Nature 2018). All data were made available to public repositories during that submission. Targeted sequencing data for the discovery cohort are deposited in the European Genome-phenome Archive (http://www.ebi.ac.uk/ega/)

under accession number EGAD00001003583. Simulated data has been made available through GitHub at https://github.com/kimskead/popgenArch. Source data for figures are provided with this paper.

Field-specific reporting

Please select the one below that is the best fit for your research. If you are not sure, read the appropriate sections before making your selection.

- [x] Life sciences
- [ ] Behavioural & social sciences
- [ ] Ecological, evolutionary & environmental sciences

For a reference copy of the document with all sections, see nature.com/documents/nr-reporting-summary-flat.pdf

Life sciences study design

All studies must disclose on these points even when the disclosure is negative.

Sample size	92 pre-AML individuals and 385 healthy controls. These individuals were the ones who had developed AML in the cohort and for whom a blood sample was available since 1993. We had designed a 4.5x matching control in data from a previously published study.
Data exclusions	None.
Replication	In silica data were used for training. Over 9 million simulations were performed.
Randomization	This is not relevant to our study. All cases were individuals who developed AML and all controls were individuals who were disease free at the time of censorship.
Blinding	Population cohort data was used where participants were followed longitudinally and captured for this study. The authors were not blinded to group allocation. These participants were part of a population cohort and we needed to identify individuals in the cohort who progressed to disease vs those which remained healthy.

Reporting for specific materials, systems and methods

We require information from authors about some types of materials, experimental systems and methods used in many studies. Here, indicate whether each material, system or method listed is relevant to your study. If you are not sure if a list item applies to your research, read the appropriate section before selecting a response.

Materials & experimental systems

n/a	Involved in the study	
☒	☐	Antibodies
☒	☐	Eukaryotic cell lines
☒	☐	Palaeontology and archaeology
☒	☐	Animals and other organisms
☐	☒	Human research participants
☒	☐	Clinical data
☒	☐	Dual use research of concern

Methods

n/a	Involved in the study	
☒	☐	ChIP-seq
☒	☐	Flow cytometry
☒	☐	MRI-based neuroimaging

Human research participants

Policy information about studies involving human research participants

Population characteristics	Population cohort data was used where participants were followed longitudinally in the EPIC cohort. All pre-AML participants were captured and 4X age and sex matched for this study. All participants were described in a previous study Abelson et al. 2018 Nature.
Recruitment	Population cohort data was used where participants were followed longitudinally in the EPIC cohort, and captured for this study. All participants were described in a previous study Abelson et al. 2018 Nature.
Ethics oversight	University of Toronto Ethics Review Board REB

Note that full information on the approval of the study protocol must also be provided in the manuscript.
